# Impact of chronic kidney disease on Watchman implantation: experience with 300 consecutive left atrial appendage closures at a single center

**DOI:** 10.1007/s00380-018-1157-x

**Published:** 2018-03-22

**Authors:** Xin Xue, Lisheng Jiang, Erich Duenninger, Manuela Muenzel, Shaofeng Guan, Adam Fazakas, Fanzhou Cheng, Juergen Illnitzky, Thorsten Keil, Jiangtao Yu

**Affiliations:** 1 Department of Cardiology, Helmut-G.-Walther-Klinikum, 96215 Lichtenfels, Germany; 2 Department of Cardiology, The Second Hospital, Jilin University, Changchun, People’s Republic of China; 3 Department of Cardiology, Chest Hospital, School of Medicine, Shanghai Jiaotong University, Shanghai, People’s Republic of China; 4 Department of Cardiology, Luohu People’s Hospital, Shenzhen, People’s Republic of China; 5 Department of Nephrology, Helmut-G.-Walther-Klinikum, Lichtenfels, Germany; 6 Department of Anesthesiology, Helmut-G.-Walther-Klinikum, Lichtenfels, Germany

**Keywords:** Atrial fibrillation, Left atrial appendage closure, Chronic kidney disease, Stroke

## Abstract

The prevalence of chronic kidney disease (CKD) is high in patients with atrial fibrillation (AF). Left atrial appendage closure (LAAC) has been recognized as an efficient alternative to oral anticoagulation for the prevention of thromboembolic events in patients with non-valvular AF (NVAF); however, the long-term safety and efficacy of LAAC in patients with CKD remain unclear. This study was designed to provide data regarding the safety and efficacy of LAAC in NVAF patients with CKD. A real-world analysis of the safety and efficacy of LAAC was performed on a cohort of 300 NVAF patients with or without CKD who underwent LAAC using the Watchman (WM) device at our center. The patients with CKD (*n* = 151) were significantly older (77.0 ± 7.2 vs. 73.2 ± 7.8 years, respectively, *P* < 0.0001) and had a higher CHA2DS2-VASc score (4.3 ± 1.5 vs. 3.4 ± 1.4, respectively, *P* < 0.0001) and HAS-BLED score (4.0 ± 1.0 vs. 3.0 ± 1.0, respectively, *P* < 0.0001) than the patients without CKD (*n* = 149). However, there were no differences between groups with respect to the device implant success rate (98.7 vs. 97.3%, respectively, *P* = 0.446) or severe periprocedural complications within 7 days. The patients were followed up for 637 ± 398 days, and all patients received repeat transesophageal echocardiography (TEE). Thirteen (4.3%) device-related thrombi, 3 (1.0%) ischemic strokes, and 19 (6.3%) non-procedural major bleeding cases were documented, and there were no differences in these complications between groups. The observed rate of all thromboembolic events by Kaplan–Meier analysis decreased by 68.8% (CKD) and 48.6% (non-CKD); moreover, the observed annual rate of bleeding was reduced by 57.5% (CKD) and 11.4% (non-CKD). Our results indicate that LAAC with the WM device is safe and effective in preventing stroke in NVAF patients with and without CKD.

## Introduction

Atrial fibrillation (AF) is the most common cardiac arrhythmia, affecting 3–5% of the population aged 65–75 years and up to 8% of individuals older than 80 years [1, 2]. The risk of stroke in AF patients increases with age, accounting for up to 30% of strokes in patients older than 80 years [3]. Oral anticoagulant (OAC) treatment is recommended for stroke prevention in AF patients [4]. Warfarin is the most common choice. However, this treatment has many limitations, including risk of bleeding, narrow therapeutic window and need for frequent INR monitoring and frequent dose adjustments [5]. New OACs (NOACs), such as dabigatran, rivaroxaban and apixaban, have recently been demonstrated to exhibit non-inferior or superior efficacy to warfarin in clinical trials [5–7] and are indicated for use in non-valvular AF (NVAF) based on the recent ESC guidelines per the ΙA recommendation [4]. However, the major bleeding risk of NOACs remains high, with reports of 2.1–3.6% annually [5–7], and the higher costs of NOACs lead to poor cost effectiveness [8].

Percutaneous left atrial appendage closure (LAAC) is an evolving therapy [9, 10]. The results of studies on PROTECT-AF demonstrate that LAAC is non-inferior and even superior to OAC therapy in the long term for the prevention of thrombotic complications [11, 12]. LAAC has subsequently been recognized in many countries as a new intervention for the contraindication of (N)OAC NVAF patients [10].

The prevalence of chronic kidney disease (CKD) is high in patients with AF. Even without a high CHA2DS2-VASc score, CKD is an independent risk factor for stroke [13]. Furthermore, CKD significantly increases the risk of bleeding, particularly when patients receive anticoagulant treatment. NOACs were approved for stroke prevention in AF; however, their use remains controversial under conditions of serious CKD, particularly for advanced or end-stage renal failure patients (eGFR < 30 ml/min/m^2^). Therefore, LAAC as a non-pharmacological method may represent a potential alternative for NVAF patients with CKD.

This study was designed to assess the procedural safety and efficacy of LAAC with the Watchman^®^ (WM) device in patients with CKD and non-CKD from a single center.

## Methods

### Patient inclusion criteria

From February 2012 to January 2017, percutaneous LAAC was performed in 300 patients with chronic or paroxysmal NVAF by implanting the WM left atrial appendage device (Boston Scientific, Marlborough, MA, USA) at our single center (Helmut-G.-Walther-Klinikum, Lichtenfels, Germany). The procedure was performed by a well trained and experienced operator and under fluoroscopy and transesophageal echocardiography (TEE) guidance. A retrospective post hoc analysis of the demographic characteristics, procedural success rate, safety and efficacy of LAAC was performed for all patients. Patients with NVAF who had a CHA2DS2-VASc score ≥ 2 or a CHA2DS2-VASc score of 1 with at least one of the following conditions were eligible: bleeding complications while using OACs, bleeding history leading to markedly elevated risk of recurrence with OAC use, HAS-BLED score ≥ 3, difficulty managing the dose of warfarin to maintain a stable INR level, or OAC refusal. The exclusion criteria included symptomatic valvular disease, symptomatic carotid disease, pregnancy, intracardiac thrombus visualized by TEE within 48 h of the planned implantation, and indication other than AF for OAC therapy.

### Renal failure definition

The estimated glomerular filtration rate (eGFR) was calculated using the Modification of Diet in Renal Disease equation [14]. According to the guidelines, CKD was defined as an eGFR < 60 ml/min per 1.73 m^2^. eGFR (ml/min per 1.73 m^2^) = 186 × (Scr) − 1.154 × age-0.203 × (0.742 if female) [15]. (Stage I: eGFR ≥ 90 ml/min, Stage II: 60 ≤ eGFR < 90 ml/min, Stage III: 30 ≤ eGFR < 60 ml/min, Stage IV: 15 ≤ eGFR < 30 ml/min, and Stage V: eGFR < 15 ml/min.)

### Procedure and follow-up

The device implantation procedure was the same as previously described in detail [16]. Briefly, LAAC was performed with the patient under general anesthesia. Intravenous heparin was administered as a bolus dose to achieve an activated clotting time (ACT) of > 250 s in all patients. TEE and an LAAC angiogram were used to determine the optimal device size (there are 5 sizes that range from 21 to 33 mm in maximum diameter). Based on IFU, all devices met the PASS (position, anchor, size, and seal) criteria prior to device release and were successfully implanted. After the operation, the patients remained in the hospital overnight and were discharged the next day following the exclusion of significant pericardial effusion/tamponade by transthoracic echocardiography (TTE) examination and the exclusion of relevant vascular complications. TEE follow-ups were scheduled at 45 days and 6 months. The post-implant drug regimen was warfarin if no contraindication or a combination of enoxaparin and aspirin if a contraindication to warfarin was present, administered for 45 days. If the TEE showed complete LAAC, no residual peri-device flow (jet > 5 mm in width), and no device-related thrombus, the patient was switched to both aspirin and clopidogrel for 6 months until the second TEE exam and then to aspirin alone.

If thrombi were detected, the anticoagulation regime was reinitiated with warfarin and aspirin until complete resolution of the thrombus by repeat TEE exam.

### Endpoints

The primary endpoint was the successful rate of LAAC. The secondary endpoints included severe complications during the periprocedural period within 7 days and major adverse events during the entire follow-up period. The severe complications and major adverse events were defined as death, stroke, transient ischemic attack (TIA), other systemic embolism, device-related thrombosis or dislocation, incomplete LAAC (gap ≥ 5 mm) and major bleeding that required invasive treatment or blood transfusion. Successful LAAC was determined by TEE, with no or minimal leak flow (gap < 5 mm). Closure was confirmed at 3 different time points: at the end of the implantation procedure, after 1.5 months and after 6 months.

To evaluate the efficacy of LAAC for the prevention of thrombosis and bleeding events, we compared the actual event rates with the predicted rate by the CHA2DS2-VASc [17] score system and HAS-BLED [18] score system according to Kaplan–Meier estimation. The total numbers of thromboembolic events (stroke, TIA and systemic embolism) and bleeding events during both the periprocedural and follow-up periods were divided by the total patient-years of follow-up and were multiplied by 100 to obtain the actual annual rate of thromboembolic events. Thromboembolism and bleeding reduction were calculated as follows: (estimated % − actual % event rate)/estimated % event rate.

### Statistical analysis

Continuous variables are presented as means ± standard deviations. Categorical variables are presented as counts and percentages. Continuous variables were analyzed using the independent samples *t* test, and categorical variables were assessed using Fisher’s exact test. Estimates for freedom from the composite of death were obtained by the Kaplan–Meier estimation method. *P* values are based on Fisher’s exact test for binomial proportions. A *P* value < 0.05 was considered statistically significant. Analyses were performed using SPSS version 24.0 (SPSS Inc., Chicago, IL).

## Results

### Baseline demographic and clinical characteristics

Three hundred patients with NVAF with long-term contraindication of OAC underwent LAAC. The baseline demographic and clinical characteristics are listed in Table 1. According to the eGFR level, 149 patients were included in the non-CKD group with normal or mild decreased eGFR (stage I and stage II); moreover, 151 patients were diagnosed with moderate or severe CKD, of which 79 patients were in stage III, 65 in stage IV and 7 in stage V. Among the patients with CKD, the average CHA2DS2-VASc and HAS-BLED scores were 4.3 ± 1.5 and 4.0 ± 1.0, respectively, which were higher than the scores in the non-CKD group. Thus, the patients with CKD had a higher risk of thromboembolism and bleeding than did the patients without CKD. Moreover, the patients in the CKD group were older and had higher rates of congestive heart failure and diabetes than the patients without CKD (Table 1).

**Table 1:** Baseline demographic and clinical characteristics

Characteristics	All	Non-CKD (stages I + II)	CKD (stages III + IV + V)	*P* value
	*N* = 300	149	151	
Age, years (mean ± SD)	75.1 ± 7.7	73.2 ± 7.8	77.0 ± 7.2	< 0.0001
Male/female, *n/n*	203/97	111/38	92/59	0.019
GFR (mean ± SD)	65.4 ± 26.0	86.7 ± 15.9	44.3 ± 14.2	< 0.0001
Permanent AF, *n* (%)	203 (67.7)	99 (66.4)	104 (68.9)	0.712
Paroxysmal/persistent AF, *n* (%)	97 (32.3)	50 (33.6)	47 (31.1)	0.712
Congestive heart failure, *n* (%)	48 (16.0)	14 (9.4)	34 (22.5)	0.003
Hypertension, *n* (%)	238 (79.3)	115 (77.2)	123 (81.5)	0.394
Diabetes mellitus (DM), *n* (%)	85 (28.3)	29 (19.5)	56 (37.1)	0.001
Stroke, *n* (%)	35 (11.7)	18 (12.1)	17 (11.3)	0.859
TIA, *n* (%)	4 (1.3)	4 (2.7)	0 (0)	0.06
CHA2DS2-VASc score (mean ± SD)	3.8 ± 1.5	3.4 ± 1.4	4.3 ± 1.5	< 0.0001
HAS-BLED score (mean ± SD)	3.5 ± 1.1	3.0 ± 1.0	4.0 ± 1.0	< 0.0001
Bleeding history, *n* (%)	86 (28.7)	47 (31.5)	39 (25.8)	0.308

*TIA* transient ischemic attack, *DM* diabetes mellitus, *CKD* chronic kidney disease

## Endpoints

### Primary endpoint

In the present study, 300 patients with NVAF underwent LAAC with the WM device. Four patients received two WM devices via a staged approach as a result of the complex anatomy of the LAA, and 6 patients had unsuccessful LAAC using the WM device because of unsuitable LAA anatomy. The overall procedural success rate was 98.0%, with no significant difference between the CKD and non-CKD groups (98.7 vs 97.3%, respectively, *P* = 0.446) (Table 2).

**Table 2:** Comparison of the success rate of LAAC between CKD and non-CKD groups

	All	Non-CKD (stages I + II)	CKD (stages III + IV + V)	*P* value
	*N* = 300	149	151	
Procedure success rate	294 (98.0%)	145 (97.3%)	149 (98.7%)	0.446

### Secondary endpoints

#### Periprocedural complications

All 300 patients who underwent LAAC were observed and analyzed for severe periprocedural complications (listed in Table 3) within 7 days. Among these patients, 10 patients (3.3%) suffered from severe complications, including 4 (1.3%) pericardial effusion/tamponade, 1 (0.3%) stroke, 4 (1.3%) device-related thrombosis and 1 (0.3%) device dislocation. No significant differences were identified in periprocedural events between the CKD and non-CKD groups.

**Table 3:** Severe complications during the periprocedural period within 7 days

Complications and adverse events	All	Non-CKD (stages I + II)	CKD (stages III + IV + V)	*P* value
	*N* = 300	149	151	
Major bleeding, *n* (%)	0	0	0	1.0
Pericardial effusion/tamponade, *n* (%)	4 (1.3)	3 (2.0)	1 (0.7)	0.369
Stroke, *n* (%)	1 (0.3)	0	1 (0.7)	1.0
Transient ischemic attack, *n* (%)	0	0	0	1.0
Other systemic embolization, *n* (%)	0	0	0	1.0
Thrombosis events, *n* (%)	0	0	0	1.0
Thrombosis with device, *n* (%)	4 (1.3)	1 (0.7)	3 (2.0)	0.623
Device dislocation, *n* (%)	1 (0.3)	1 (0.7)	0	0.497
Device-related death, *n* (%)	0	0	0	1.0
Total, *n* (%)	10 (3.3)	5 (3.4)	5 (3.3)	1.0

The patients who suffered from pericardial effusion/tamponade during LAAC avoided surgical operation because of timely pericardial puncture. Of the 4 cases of device-related thrombi occurring during LAAC, 3 cases occurred in the access sheath; these cases were removed by washing with water and adding a heparin dose. The other case of thrombosis, which occurred on the surface of the device, was recurrent despite attempts to dissolve the thrombus by increasing the dose of heparin and using a platelet glycoprotein IIb/IIIa receptor antagonist; thus, device placement was abandoned (Table 3).

#### Follow-up

In the mean follow-up period of 637 ± 398 days, 212 (70.4%) patients had a follow-up period greater than 365 days, and 524 patient-years (274 patient-years in the non-CKD group and 270 in the CKD group) were collected. All patients (100%) received at least one TEE exam at 6 weeks after the operation and clinical follow-up to monitor major adverse events through outpatient or telephone assessments.

Among the 300 patients, 11 (3.7%) thrombotic or thromboembolic events were identified, including 3 (1.0%) ischemic strokes and 8 (2.7%) TIAs; 3 patients experienced both TIA and stroke. Nineteen (6.3%) patients suffered from major bleeding events, including 4 (1.3%) cerebral hemorrhages, 10 (3.3%) gastrointestinal bleedings and 5 (1.7%) other bleedings, however, 58 (19.3%) patients had a complex clinical status, including a pacemaker, wound, surgical operation or tumor, which present a high risk of bleeding. Thirty-five (11.7%) all-cause deaths were identified (from February 2012 to January 2017), including 32 (10.7%) deaths in patients with successful LAAC and 3 deaths in patients without successful LAAC. However, only 5 (1.7%) deaths were proven to be cardiac related, and no device-related deaths were identified during the entire follow-up period. By TEE follow-up, 13 (4.3%) device-related thrombi and 2 (0.7%) gaps (> 5 mm) between the device and the left appendage wall were identified. However, with the exception of the higher incidence rate in patients in the CKD group with a complex clinical status with a high risk of bleeding (6.3 vs 25.8%, respectively, *P* = 0.005), there was no significant difference in the major adverse events between the CKD and non-CKD groups (Table 4).

**Table 4:** Major adverse events during follow-up

Complications and adverse events	All	Non-CKD (stages I + II)	CKD (stages III + IV + V)	*P* value
	*N* = 300	149	151	
Thrombosis event, *n* (%)	11 (3.7)	8 (5.4)	3 (2.0)	0.137
Ischemic stroke, *n* (%)	3 (1.0)	2 (1.3)	1 (0.7)	0.621
Transient ischemic attack, *n* (%)	8 (2.7)	6 (4.0)	2 (1.3)	0.172
Systemic thromboembolism, *n* (%)	0	0	0	1.0
Device-related thrombosis, *n* (%)	13 (4.3)	8 (5.4)	5 (3.3)	0.411
GAP (> 5 mm), *n* (%)	2 (1.3)	2 (0.7)	0	0.246
Major bleeding, *n* (%)	19 (6.3)	9 (6.0)	10 (6.6)	1.0
Cerebral hemorrhage, *n* (%)	4 (1.3)	2 (1.3)	2 (1.3)	1.0
Gastrointestinal bleeding, *n* (%)	10 (3.3)	4 (2.7)	6 (4.0)	0.75
Other bleeding, *n* (%)	5 (1.7)	3 (2.0)	2 (1.3)	0.683
Other clinical status, *n* (%)	58 (19.3)	19 (6.3)	39 (25.8)	0.005
Pace maker, *n* (%)	18 (6.0)	6 (4.0)	12 (7.9)	0.224
Tumor, *n* (%)	7 (2.3)	2 (1.3)	5 (3.3)	0.448
Wound, *n* (%)	17 (5.7)	7 (4.7)	10 (6.6)	0.619
Operation, *n* (%)	16 (5.3)	4 (2.7)	12 (7.9)	0.069
Total all-cause death/total patients, *n* (%)	35 (11.7)	12 (8.1)	23 (15.2)	0.071
Death/successful LAAC, *n* (%)	32 (10.7)	11 (7.4)	21 (13.9)	0.091
Cardiac death/successful LAAC, *n* (%)	5 (1.7)	2 (1.3)	3 (2.0)	1.0
Non-cardiac death/successful LAAC, *n* (%)	27 (9.0)	9 (6.0)	18 (11.9)	0.105
Device-related death/successful LAAC, *n* (%)	0	0	0	1.0

**Fig. 1:**
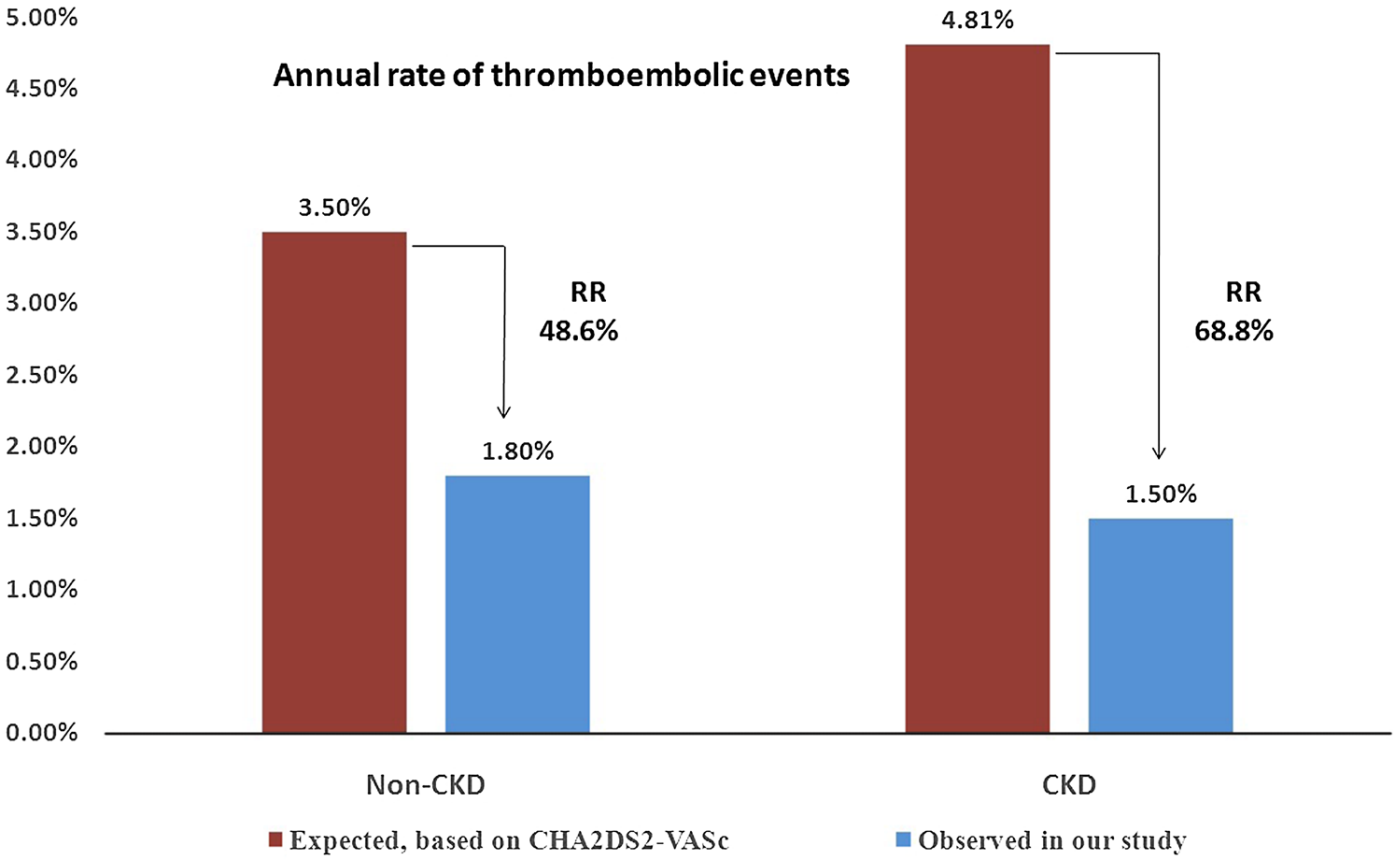
Observed annual rate of thromboembolic events, including stroke, TIA and other systemic thromboembolism vs the expected rate based on the CHA2DS2-VASc score; *RR* relative risk

**Fig. 2:**
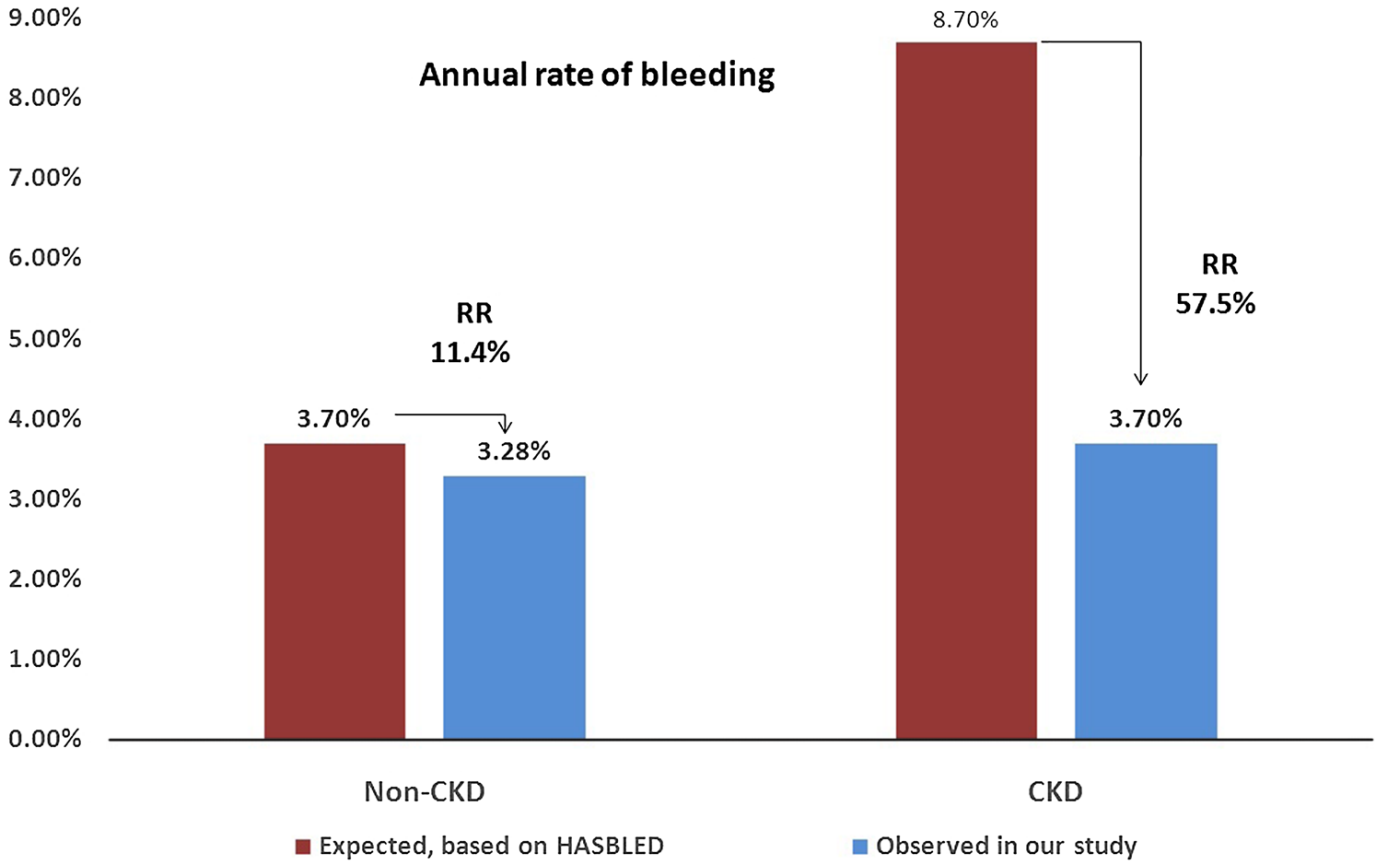
Actual annual rate of bleeding events vs expected rate based on the HAS-BLED score; *RR* relative risk

According to the Kaplan–Meier estimation, compared with the expected value based on the CHA2DS2-VASc score, the observed annual rate of thromboembolic events, including stroke, TIA and other systemic thromboembolism, decreased by 68.8% in the CKD group and 48.6% in the non-CKD group (Fig. 1). Furthermore, the observed annual bleeding rate compared to the expected bleeding rate based on the HAS-BLED score decreased by 57.5 and 11.4% in the CKD and non-CKD groups, respectively (Fig. 2).

## Discussion

In the present study, we provided real-world data regarding LAAC in 300 NVAF patients, including 151 patients with CKD and 149 patients without CKD in our single center. Our results showed that patients with CKD were older and had substantially higher CHA2DS2-VASc and HAS-BLED scores, which represent higher risks of stroke and major bleeding than patients without CKD; however, there were no significant differences in the device implant success rate or the incidence rate of severe complications in the 7-day periprocedural period between the patients with and without CKD. During the average follow-up period of 637 days, the CKD group had higher mortality; however, there were no significant differences in major adverse events, including ischemic stroke and major bleeding events, between the groups. Joelle Kefer et al. [19] also presented similar conclusions in a similar study using the ACP device; their study demonstrated that the procedural safety and efficacy of LAAC with the ACP device on stroke reduction are similarly high in patients with CKD and patients with normal renal function, with no impact of the CKD stages on the periprocedural MAEs.

Of the 300 patients who received LAAC, 6.3% patients suffered from major bleeding events, including 4 (1.3%) cases of cerebral hemorrhage, 10 (3.3%) gastrointestinal bleedings and 4 (1.7%) other bleedings. Established literature indicates that CKD increases bleeding risk. In our study, patients with CKD had complex clinical conditions that may have led to bleeding, including pacemaker implantation, wound, cancer and previous operations; however, compared with patients without CKD, the risk of major bleeding did not increase. This may be because LAAC avoids long-term OAC therapy, which easily increases the bleeding risk.

According to Kaplan–Meier estimation, compared with the expected values based on the CHA2DS2-VASc score, the observed annual rate of thromboembolic events, including stroke, TIA and other systemic thromboembolisms, decreased by 68.8% in the CKD group and 48.6% in the non-CKD group; the observed annual bleeding rate compared to the expected bleeding rate based on the HAS-BLED score was reduced by 57.5 and 11.4% in the CKD and non-CKD groups, respectively. According to our results, CKD did not reduce the efficacy and safety of LAAC; rather, patients with CKD may benefit more from LAAC in preventing thrombosis and decreasing bleeding compared with the expected risk. In clinical practice, it is difficult to determine appropriate OAC dosages for CKD patients. LAAC may be an ideal choice to prevent stroke and other thrombotic complications in NVAF with CKD.

During the follow-up period, thrombus formation on the surface of the device was detected in 13 patients via TEE; however, there were no significant differences between patients with and without CKD. We determined that thrombus formation on the surface of the device may be associated, in part, with irregular anticoagulant treatment after LAAC. Among the 13 patients with device-related thrombosis, 4 patients had device-related thrombosis at 1.5 months as a result of a low cognitive ability and alcohol addiction, which were speculated to be linked to non-compliance with the anticoagulation regimen. Nine patients were identified at 6 months during treatment with aspirin and clopidogrel; two thrombi disappeared after switching from aspirin and clopidogrel to aspirin and ticagrelor, whereas the remaining seven thrombi disappeared after the addition of anticoagulant therapy with subcutaneous enoxaparin for an additional month. The reasons for device-related thrombosis formation remain unclear but may include multiple factors [20]. Consistent with Lempereur et al. [20], we determined that the majority of the device-related thrombi were located at the device adjacent to the left superior pulmonary vein and central hub regions. We speculate that the central hub may have a delayed endothelialization response as a result of the connecting point slightly protruding. It may also be a reasonable explanation that the blood flow velocity in the area adjacent to the pulmonary vein is lower than that in the area next to the mitral valve.

Oral anticoagulation therapy is an IA recommendation [4] to prevent stroke and other thromboembolic events in AF patients. The prevalence of CKD is high in patients with AF. Moreover, CKD is not only an important risk factor of stroke [13] but also an independent predictor of increased risk of major bleeding in patients with AF [21]. In patients with CKD, regular monitoring of renal function is, therefore, required for OAC administration, and special concern related to bleeding risk, particularly cerebral and gastrointestinal bleeding, is present [22, 23]. When the eGFR is < 30 ml/min/m^2^, the NOAC dosage must be reduced or terminated [24]. Therefore, long-term oral anticoagulation therapy for AF patients with CKD is not an ideal choice. Our study indicated that the WM as a non-pharmacological method may be an alternative for NVAF patients with CKD who have high risks of stroke and major bleeding.

## Conclusions

In summary, the procedural success rate of LAAC with the WM device and the safety during the periprocedural period were similar in patients with and without CKD. During long-term follow-up, there were no significant differences in major adverse events, including death, ischemic stroke and major bleeding, between patients with and without CKD. According to Kaplan–Meier estimation, patients with CKD may benefit more from LAAC to prevent thrombosis and decrease bleeding compared with the expected risk. Thus, LAAC may be an ideal choice to prevent stroke and other thrombotic complications in patients with NVAF with CKD.

### Study limitations

Our study has several limitations. First, this study is a retrospective post hoc analysis of real-world treatment at a single center. All clinical events were obtained by telephone, outpatient visit or case histories, and the follow-ups, including medical treatment and TEE imaging, lacked standardization. Moreover, although the only operator in our study is well trained and high skilled, the large volume of LAAC procedures indicates that the conclusions of our study may not be extrapolated to other centers.
